# Prevalence and Predictors of Non-Suicidal Self-Injury among Children with Autism Spectrum Disorder

**DOI:** 10.12669/pjms.335.12931

**Published:** 2017

**Authors:** Bushra Akram, Mehak Batool, Zeeshan Rafi, Abrar Akram

**Affiliations:** 1 Dr. Bushra Akram, PhD. Assistant Professor, Department of Psychology, University of Gujrat, Gujrat, Pakistan; 2 Mehak Batool, BS Hon’s., Student at Department of Psychology, University of Gujrat, Gujrat, Pakistan; 3 Zeeshan Rafi, M.Phil. PhD Scholar, Faculty of Computer Sciences and Management, Istanbul University, Turkey; 4 Abrar Akram. MSc. Faculty of Computer Sciences and Management, Gujrat University, Gujrat, Pakistan

**Keywords:** Prevalence, Non Suicidal self-injury, Autism Spectrum Disorder, Self-Injurious Behavior

## Abstract

**Objectives:**

To find the prevalence as well as to identify the predictors as protective and risk factors of Non-Suicidal Self-Injury (NSSI) among children with autism spectrum disorder (ASD).

**Methods:**

In this analytical cross sectional survey 83 children with ASD age range from 8 to 18 years were selected through convenient sampling technique from five special schools of Lahore city. The Urdu form of a standardized tool was used to assess NSSI.

**Results:**

Statistical analysis indicated overall point prevalence of NSSI was 33%. Moreover banging/self-beating (47%), scratching (38), pinching (35%), picking scabs (33%), self-biting (32%), pulling hair (30%) and rubbing skin (19%) emerged as common forms of challenging behavior. Further regression analysis showed that age B(1.68*, P<.05), gender B(3.72, P<.001) and severity level of ASD B(1.85***, p<.0001) as risk factors/positive predictors of NSSI. However early intervention (-0.66***, P<.0001) and involvement of parents in counselling (-2.66*, P<.05) emerged as protective factors/negative predictors of NSSI among children with ASD.

**Conclusion:**

Non-suicidal self-injury is a serious challenge among children with ASD. Early intervention, counselling and parental involvement in managing the children with ASD will not only prevent but reduce the challenging behaviors.

## INTRODUCTION

Autism Spectrum Disorder (ASD) is a neuro-developmental disorder. The individuals with ASD show deficits in social functioning, language, communication skills and ability to maintain relationships. It is a spectrum disorder which means that its symptom patterns range of abilities and characteristics are expressed in many different combinations and in any degree of severity. They are also found to exhibit unusual interest and stereotype behaviors.^1^

Self-injurious behaviors (SIB) are self-inflicted behaviors that are harmful for the child’s own body and these include head banging, hand or arm biting, excessive scratching and rubbing, self-choking, hair pulling and many others.^2^-^4^ Researchers report high rate of physically dangerous challenging behavior among children with ASD. They further indicated lack of awareness and sincere efforts for early diagnosis and intervention on the part of stake holders.^2^,^5^,^6^ It has been indicated that the children with ASD, observed to be at greater risk of developing SIB as compared to the individuals with sensory, language impairments and those with other medical problems like headaches, seizures and other infections.^5^-^7^

There are some studies which showed the prevalence of SIB 30% or higher in the population with ASD.^6^ The results of longitudinal researches showed that almost 50% of the individuals with ASD involve in some form of self- injury in any specific time period of their life span.^8^ Another study reported history of non-suicidal self-injury.in 50% of the participants with ASD. Moreover females with ASD showed significantly higher self-injury as compared to their male counterparts.^5^ Similarly 84% of the participants with intellectual disabilities continued to show self-injurious behavior even 20 years later with no significant changes in the type and severity. Majority of them had ASD as an additional disability.^9^ On the other hand a few studies found head banging, eye poking and self-hitting in one year children with ASD.^10^ Another study reported 27.7% prevalence of SIB among a large sample of children with ASD in USA. However the prevalence of SIB predicts very poor long term outcomes for the individuals with ASD.^2^,^3^

Though prevalence of self-injury without the intentions of suicide is common in ASD but it is not considered the symptom of ASD because self-injury has been recorded in the individuals’ with other disabilities as well as in the normal population. Self injurious behaviors is assumed to be a repetitive behavior at the same time it can be episodic as it either occurs under highly specific stimulus contexts, or in bursts after long periods without problematic behavior.^8^,^11^

### Rationale of the study

There is a dearth of published studies on NSSI of individuals with ASD in Pakistan in spite of the fact that SIB is highly associated with ASD. Self-injury is a source of frustration to parents, caregivers, teachers and other professionals working with these children. The present study examined the point prevalence of NSSI among children with ASD and can be a source of awareness to the parents, teachers and other stakeholders. The results also identify the role of demographics and other environmental factors contributing as risk as well as the protecting factors of developing NSSI. This study can be helpful to design preventive measures to control the NSSI among children with ASD by indicating the protective and risk factors.^6^,^12^,^13^

The main objectives of this study were to:

- Find prevalence of Non suicidal self-injury among children with ASD across forms of NSSI and across the personal characteristics of the participants.
- Explore the common forms of self-harm behaviors and identify the risk as well as protective factors of NSSI among the children with ASD.

## METHODS

Total 95 children with ASD were conveniently selected from the five institutions located in Lahore city. Their mean age was 11.77 (SD, 3.59). Of them 60 were male and gender mean was 1.36 (SD, 0.48). The detail of the personal characteristics of the participants are given in Table-I. Their parents were accessed to complete Urdu^14^ form of a standardized Inventory of statements about self-injury (ISAS).^15^ The first section of the tool measured the prevalence and forms of SIB behavior whereas second section assesses prevalence of NSSI among the participants with ASD. ISAS was completed by the parents of 83 children. Internal consistency of the tool in the present study is 0.86.

**Table-I:** Prevalence of NSSI among participants across their personal characteristics in percentages.

		*Not relevant (%age)	**Somewhat relevant (%age)	***Very Relevant (%age)
Overall prevalence	F (%)	27.7	39.3	33.0
***Gender***				
Male	57(68)	61.5	40	41.9
Female	35(32)	38.5	60	58.1
***Age***				
5-10	31(37)	30.8	38.5	25.8
11-15	30(36)	30.8	48.7	45.2
16-20	22(27)	38.4	12.8	29.0
***Severity level of ASD***
Mild	24(29)	61.5	10.3	06.5
Moderate	27(32)	30.8	33.3	25.8
Severe	32(39)	7.7	56.4	67
***Parental involvement in counselling***
Yes	32(38)	30.8	71.8	74.2
No	51(62)	69.2	28.2	25.8
***Early Intervention***
Yes	35(40)	76.9	36.5	20
No	50(60)	23.1	64.5	80

* Not relevant means no NSSI

** Somewhat relevant indicates mild and

*** very relevant means moderate to severe NSSI.

The assessment process was carried out at two phases. At first phase the management of the schools pointed 132 children with ASD. At the second phase the childhood autism rating scale (CARS)^16^ was implemented to confirm the diagnosis of these 132 children. In the result 95 children appeared to have Autism Spectrum Disorder as a primary diagnosis. The rest of the children were found with co-morbidities therefore they were deleted from list. Therefore total 83 children were included in the study.

The study was approved by departmental review board. Informed consent was taken from the heads of the institutions and the parents of the participants. The participants were told that they can quit from the study at any stage.

## RESULTS

In order to find the prevalence and forms of self-injurious behavior among children with ASD frequency program was run and percentages were calculated. The detail is given in Table I and II.

**Table-II:** Prevalence of forms of SIB in percentages among the participants.

*Forms of SIB*	*Prevalence in percentage (%)*
Banging or self-beating	47
Severe scratching	38
Pinching	35
Interfering with wounds healing(e.g., picking scabs	33
Biting	32
Pulling hair	30
Rubbing skin against rough surface	19
Cutting	02
Swallowing dangerous substances	02
Sticking self w/Needles	01
Burning	0.5
Carving	0

The prevalence of forms of self-injurious behaviors in percentages among children with ASD is shown in Table-I. Majority of the participants (47%) show banging or self-beating as self-harm behavior whereas scratching (38%), pinching (35%), picking scabs (33%) biting (32%) pulling hair (30%) and rubbing skin against rough surface (19%) are other common self-injurious behaviors. On the other hand cutting, swallowing dangerous substances, sticking self with needles and burning are less common behaviors. Moreover the participants with ASD of present research have not reported carving with stone at all.

Overall prevalence of NSSI among the participants and the prevalence of NSSI among children with ASD according to associated characteristics is shown in Table-II. Overall Moderate to severe prevalence of NSSI has been observed up to 33% whereas mild prevalence is 39.3%. Female appeared to be more exhibit Non-suicidal self-injury behavior as compared to their male counter parts. It is also observed that the adolescent participants are more involved in NSSI as compared to other age groups. Further the children with ASD whose parents participate in the process of counselling show less self-harm behavior. Similarly the participants who received early intervention for their symptoms of ASD exhibit less Non suicidal self-injury as compared to other group who did not receive any services in their early years. Early intervention refers to the timely services such as assessment and treatment to the very young children with or without special needs. Generally these services are given to the children from birth to three years.

It is further supported by the results of multiple regressions Table-III where severity of the autistic symptomatology emerged as a significant risk factor/predictor of NSSI just like the previous researches reported the positive relationship between the frequency and intensity of SIB with the severity level of ASD among the individuals. Regression analysis was run to find the predictors as protective and risk factors of NSSI.

**Table-III:** Standard Multiple linear Regression analysis on the study variables as predictors of NSSI.

	*B*	*SEB*	*Β*
Constant	22.49	3.32	
Age	1.68*	0.69	0.24
Gender	3.72**	1.12	0.32
Severity level of ASD	1.85***	0.51	0.31
Parental Involvement in counselling	-2.66*	1.25	-0.29
Early Intervention	-0.66***	0.18	-0.14
∆R^2^	0.88		
F	49.71***		

In order to identify the predictors of NSSI among the participants with ASD standard multiple regression was run on the data. The values of ∆R^2^ (0.88) and *F* ratio (49.71, *p* <.001) show the goodness of fit of the model. It means that the independent variables are explaining 88% of the variance in dependant variables. In the result age, gender, severity level of ASD, parental involvement in counselling and early intervention emerge as significant predictors of NSSI. However Age and severity level of ASD are the positive and turned out as risk factors. On the other hand early intervention and parental involvement in the management plans of the children emerged as protective factors and negative predictors of NSSI.

## DISCUSSION

The scarcity of research on prevalence of NSSI among children with ASD in Pakistan was the motive to conduct present study. Although a fair number of studies investigated the prevalence of SIB among the population with intellectual disabilities but there is a limited published data describing challenging behaviors of ASD. The previous studies highlighted the need of identifying the associated factors of self-injury among ASD so that the challenging behaviors may be stopped.^1^

The exact reasons of these stereotype self-stimulatory injurious behaviors are not known. However ASD limits the functioning of many parts of the brain which further affects every aspect of social interactions with others and weakens the abilities like social responsiveness, communication and feelings for other people.^2^ The children with ASD have been found to engage in extreme and sometimes potentially life threatening self-injurious behaviors.

The results of frequencies in the form of percentages in this study indicates that majority of the participants showed head banging or self-beating, scratching, pinching, picking scabs, biting, pulling hair and rubbing skin against rough surface as self-injurious behaviors. These findings are consistent with the results of previous studies.^1^-^4^ Majority of children were exhibiting these behaviors since their early childhood and they show this behavior daily for more than once.^2^,^3^

Results of present study indicate 30% overall prevalence of NSSI among the participants. The findings of the researches conducted in west indicates up to 50% prevalence of SIB among individuals with ASD.^2^-^4^,^6^ Another study gave evidence of 52.3% of the children to indicate SIB.^12^ Similarly a recent study indicated the prevalence of 35% of SIB in children with ASD.^17^

Further the comparison across personal characteristics indicated that females appeared to be scored higher on non-suicidal self injurious behaviors as compared to their male counterparts. A reason of these gender differences can be that girls with ASD were found to show more cognitive developmental delays and behavioral problems as compare to the boys with similar disorder.^18^ These findings are in line with other studies where girls with ASD were found to report more NSSI as compare to boys.^2^ The result of present study also showed that the rate of NSSI varies across age groups. Adolescents reported higher rate of self-harm behavior as compared to the individuals in other age groups which has also been supported by the results of a recent study.^6^,^12^ A plausible reason of this increase may be that SIB is associated with the physical and hormonal changes occur in this developmental period that also bring challenges to even the adolescents without ASD.^3^

Present study also found the highest prevalence of NSSI among the participants who had severe level of ASD. It means the severe the level of symptoms of ASD the more frequent and severe SIB among the individuals. Similarly researcher^6^ suggest that SIB increases as the degree of disorder and inability to maintain daily living get worsened. Another study concluded the severity of symptoms of ASD is a risk factor of SIB.^6^,^19^,^20^ It is evident that the participants with more cognitive and developmental deficits may exhibit increased severity of symptoms.^18^ It has also been explored that children who receive intervention are less likely to get involve in self-injurious behavior than others. Other behavioral Intervention for autism spectrum disorder has found to be beneficial in terms of self-injurious behavior in other studies as well.^4^,^21^,^22^,^23^ Studies report an improvement in cognition, communication and behavior of young children with ASD due to early intervention.^19^,^20^ It was observed that children whose parents were got involved in the counseling session to deal with ASD showed less self-injurious behavior. Studies report an agreement with these finding and indicate reduced ASD symptomology due to parental intervention.^21^,^22^ The results of this study provide promising implications for parents as well as practitioners who work with young children with challenging behaviors. Parent involvement with their children with challenging behaviors is critical.^21^ Since parents spend a significant amount of time with their children, collaborating with parents to design interventions is a promising approach to help reduce children’s challenging behaviors.^3^,^22^,^24^

The findings of regression analysis supported the above mentioned statements hence parental involvement in the process of counseling and provision of early intervention as protective factors as well as the severity of ASD is a major risk factor of NSSI among the children with ASD.^6^,^19^,^21^

### Limitations

The main limitations of the study was small sample size selected from the Lahore city due to time and financial constraints. The more generalizable results would have been generated in case of recruiting the sample from other regions also.

## CONCLUSION

The present study found some associated factors as predictors of Non-suicidal Injury among children with ASD which further may help to tailor preventive and intervention programs to reduce the life threatening challenging behaviors. Early interventions, the focus counselling in adolescence and involvement of parents/caregivers in the treatment or management plans of the individuals with ASD will reduce the severity of symptoms which will further help in minimizing the challenging behaviors.

### Authors’ Contribution

**BA** conceived, designed and editing of manuscript.

**MB, ZR, AA,** did data collection, statistical analysis and manuscript writing.

**BA** takes the responsibility and is accountable for all aspects of the work in ensuring that questions related to the accuracy or integrity of any part of the work are appropriately investigated and resolved.
